# Adapting emotional support in teams: productivity, emotional stability, and conscientiousness

**DOI:** 10.3389/frai.2025.1449176

**Published:** 2025-03-28

**Authors:** Isabella Saccardi, Judith Masthoff

**Affiliations:** Department of Information and Computing Sciences, Utrecht University, Utrecht, Netherlands

**Keywords:** emotional support, groups, personalization, collaborative learning, personality

## Abstract

Students' mental health has received increased attention in recent years: reports of worsened mental health among higher education students call for new ways to support them in their college years. Educational demands are among the concerns that students report, such as the stress of academic performance, the stress related to examinations and the pressure to succeed. One aspect often present in higher education is group work. Group work can be truly beneficial for learning, but it often causes additional stress to students. The present research contributes to the design of a peer assessment tool to support students during group work. In this tool, each student is asked to rate their teammates on several aspects of group work, and a virtual agent delivers support statements in response to such ratings. For the support statements to be appropriate, the virtual agent should adapt them to the recipient and the group work situation they are experiencing. We investigate the adaptation of emotional support statements to the student's personality trait of Conscientiousness and the score assigned to a teammate on one aspect of teamwork, Productivity. The resulting algorithm is then combined with related work on Emotional Stability, and a final algorithm considering both dimensions is created.

## 1 Introduction

Starting one's college years often marks an important transition to adult life, characterized by a new independence: many move to another town and start living independently, learning to manage finances autonomously and creating a new social environment while facing new academic demands. Despite the personal growth this phase may bring, many students experience a marked worsening in their mental health (Oswalt et al., 2020; Robotham, 2008; Pedrelli et al., 2015). Increasing reports of mental illnesses have brought attention to the need for better support for students in higher education. Support can take the form of improving the university's mental support services but also improving the experience itself linked to academic demands. This work focuses on the latter case by contributing to the design of a tool for supporting students working in groups.

Group projects are widely used in higher education because of their numerous benefits for learning; however, students often report negative experiences of group work (Burdett, 2003; Barfield, 2003; Pfaff and Huddleston, 2003). Ensuring a positive group work experience is a complex problem, entailing several challenges: how can the teachers know how all groups are doing when they are often responsible for many students? How do they ensure that every single student receives appropriate help based on their unique situation? A large number of students often makes it impossible for the teachers to monitor, support, and intervene when necessary. Technology may be the solution. Among many computer-supported interventions to support students, peer assessment surveys have been proposed mainly as a way to improve grade fairness (Freeman and McKenzie, 2002; Murray and Boyd, 2015; Badea and Popescu, 2019). Yet, a peer assessment survey can also be used to monitor group well-being, detect group-related issues, and support students. An example is the survey by Saccardi et al. (2023), delivered several times during a group project, to detect group problems as soon as they arise and make the students feel supported as they are filling it out, by providing appropriate support messages. The choice of support messages, however, presents a few challenges: how to ensure that a person is receiving the correct kind of support, suitable to their personality and experience of group work? *Adaptation* may be the answer, as people are known to naturally adapt emotional support messages to the personality and situation that the recipient is facing (Smith et al., 2015; Dennis et al., 2013b,a; Kindness et al., 2017).

In this paper, we present a series of studies to inform the choice of support statement for the peer assessment tool designed by Saccardi et al. (2023), where each student rates their teammates on five dimensions of teamwork. In particular, we focus on one aspect of teamwork among the five proposed by Saccardi et al. (2023), *Productivity*, the quantity of work provided for the group project. Specifically, we investigate which supportive feedback to provide to a student rating another one on Productivity, based on the score provided and the rater's personality on two traits, Conscientiousness and Emotional Stability.

## 2 Related works

In this section, we first summarize the related work regarding students' need for support, and how technology may help. Then, we present a brief exploration of the concept of emotional support and its benefits for students and the class. Thirdly, we discuss what it means to deliver appropriate support messages and the relevance of personality traits to adapt such messages. Lastly, we present the use case the present work focuses on.

### 2.1 Students' mental health crisis

Higher education students report higher levels of stress, anxiety, and depression compared to their peers (Blanco et al., 2008; Lovell et al., 2015; Ibrahim et al., 2013; Beiter et al., 2015; Hill et al., 2020). The concern for their mental health has been increasing especially with the COVID-19 pandemic, which witnessed another marked worsening in anxiety and mood disorders in this population (Buizza et al., 2022; Hamza et al., 2021). Many efforts have been directed at identifying the causes underlying this phenomenon. The changes that a student is confronted with are many and involve numerous aspects of their academic, professional, and personal life (Maunder et al., 2013; Brougham et al., 2009). Academic performance, pressure to succeed, and stress about post-graduation plans are common concerns mentioned by students in this phase (Beiter et al., 2015; Brougham et al., 2009; Robotham, 2008; Byrd and McKinney, 2012).

### 2.2 Technology supporting students

The evidence about students' mental health crisis calls for a new type of support from educational institutions—a support that targets the challenges students face and facilitates the transition to college life. Colleges typically offer mental health support services, but they are often not enough: they cannot keep up with the high number of students seeking help (Auerbach et al., 2016), they are not known by students, or students hesitate to reach out to them because of personal considerations or distress (Oswalt et al., 2020; Yorgason et al., 2008; Rosenthal and Wilson, 2008; Storrie et al., 2010).

A growing body of evidence examined the possibility of using technology-based intervention to facilitate seeking help and supporting students. Technology-based interventions may provide an alternative, more accessible way to improve students' wellbeing. Such interventions use computer or web-based interfaces to deliver mental health prevention or treatment, and they have the potential to help identify, prevent and treat mental health conditions. Several possible interventions have been explored with promising results; examples include mindfulness-based and cognitive behavioral therapy-related interventions (Lattie et al., 2019; Harrer et al., 2019; Davies et al., 2014; Conley et al., 2016, 2017). Cognitive behavioral therapies based interventions, especially, were found to effectively reduce symptoms of depression, anxiety and stress, with effects sustained over time (Conley et al., 2016, 2017). While such large-scale interventions are extremely important, it is equally necessary to work on improving the overall higher education experience, since academic demands are indeed amongst the primary sources of concern for students (Beiter et al., 2015; Brougham et al., 2009). Certain aspects of the academic world cannot be avoided; for example, the stress related to examinations, the pressure of studying, etc. However, other aspects can be improved while considering students' well-being: one is *group work*.

### 2.3 Group work as a support opportunity

Group work is ubiquitous in higher education. It has been increasingly used to provide students with valuable skills in the workplace, such as teamwork, organizational and communication skills, but also to foster learning (Hammar Chiriac, 2014; Colbeck et al., 2000; Laal and Ghodsi, 2012; Bravo et al., 2019). However, despite its benefits, students do not always welcome group projects. Tensions can arise between group members for a variety of reasons, such as group dynamics, the pressure of the group assessment and the challenge of organizing work in groups (Burdett, 2003; Barfield, 2003; Pfaff and Huddleston, 2003). Personal characteristics also play a role: the attitudes and expectations with which a student approaches teamwork can impact motivation and participation (Barfield, 2003; Mackie and Goethals, 1987). Diversity in culture, gender, age, education, and social influence may also cause the creation of sub-groups within the group and ultimately undermine team cohesion and working process (Tost et al., 2013; Garandeau et al., 2014; Homan, 2019). Unequal contributions may result in social loafing—the tendency of an individual to lower their productivity when working in a group - ultimately hindering group motivation and achievements (Ringelmann, 1913; Ingham et al., 1974; Simms and Nichols, 2014). These issues may result in communication issues, or communications issues can exist by themselves and worsen overall group performance (Cervone, 2014; Lolli, 2013). Many of these problems are interrelated (Roberts and McInnerney, 2007) and can occur simultaneously, highlighting the necessity to detect them and act as soon as possible. However, this is often not feasible for teachers, who usually deal with a large number of students and cannot closely monitor each of the working groups.

Technology interventions addressing this issue have been proposed, especially focusing on improving grade fairness using peer-based assessments (Freeman and McKenzie, 2002; Murray and Boyd, 2015; Badea and Popescu, 2019). The present study is based on the work by Saccardi et al. (2023), who developed a peer assessment survey to be administered several times during a course - allowing for earlier detection of group issues. In this tool, each student is asked to rate their teammates on five aspects of teamwork: Quality of Cooperation, Quality of Contribution, Productivity, Friendliness, and Reliability. The scores assigned to each teammate were then used to signal potentially problematic situations among groups to the teachers. The survey has been validated in a university course and was well received by both students and teachers (Saccardi et al., 2023). The primary objective of this peer assessment was to monitor groups of students and obtain an overview of the groups' well-being. At the same time, it is a survey that reaches out to every single student, opening the possibility of using it to provide support as the students fill it out. The present study develops an algorithm that can be used as part of this survey so that supportive statements are provided automatically throughout the peer assessment, contributing to the feeling of being individually seen, heard, and supported during the group project.

### 2.4 Teachers' emotional support

Detecting group problems as soon as they arise is fundamental in supporting students' group work; however, it could not be enough to ensure students feel supported throughout the course. Consider the following example, based on the case study of Saccardi et al. (2023). A bachelor course of 100 students includes a group project; students are divided into 25 teams of four people. After a few weeks, the teacher sends out a peer assessment survey; this reports five groups potentially facing a problem, who are contacted by the teacher. These groups will likely feel supported, but will the remaining groups feel the same? Reaching out when a problem is found is necessary; ensuring that the rest of the class feels supported is equally important.

The concept of support is a widely researched topic in psychology. It constitutes an essential aspect of social relationships: it is crucial to build a healthy social circle, but it is also important in an academic and professional setting to foster a positive and healthy environment (Jayaratne et al., 1988; Harris et al., 2007; Titsworth et al., 2010, 2013). Amongst the many forms that social support can take, *emotional support* can be defined as communicative behavior directed from one party to another with the intent of helping the other cope with negative emotions (Burleson, 2003). Emotionally supportive communication includes expressions of appreciation, care and encouragement toward the distressed party (Burleson and Goldsmith, 1996; Thoits, 1995). Sources of emotional support can be closer friends or family, but emotional support often addresses everyday stressors and worries and can also be effective when delivered by colleagues, less close friends, or teachers (Burleson, 2003; Romano et al., 2020). Teachers' emotional support, especially, has gained a renewed interest for its potential to increase motivation and decrease behavioral issues in middle and high school students (Yeung and Leadbeater, 2010). Although the research on teachers' emotional support in higher education is limited, there is evidence of its benefit in improving positive effects and learning outcomes in college classes (Titsworth et al., 2010, 2013). In this context, teachers' perceived emotional support is defined as the perceived availability to provide support about topics directly and indirectly related to school, and it is closely related to their capability to provide a warm and positive communication style.

**Figure 1:**
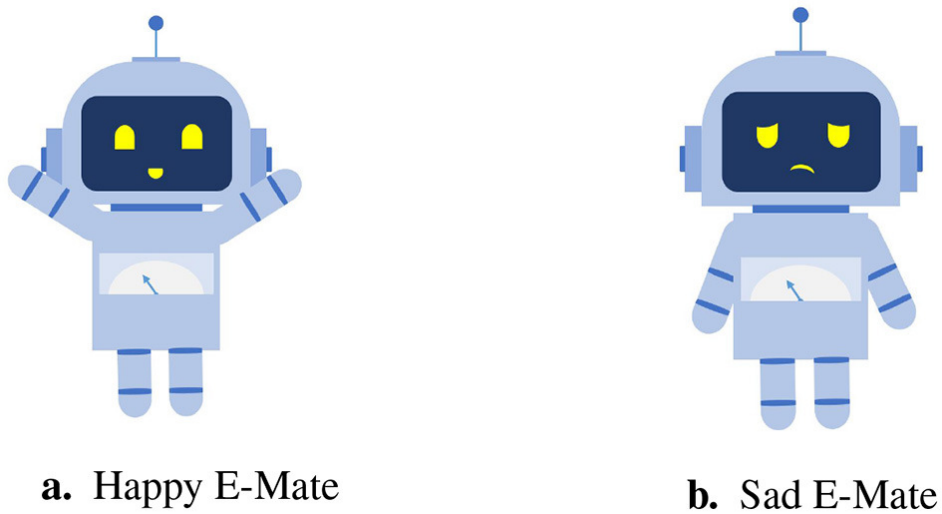
E-Mate examples of happy **(a)** and sad **(b)** reactions reproduced from Saccardi et al. (2023).

Given this definition of teachers' emotional support, it is therefore evident how it is not enough to reach out when a problem is found, but it is rather necessary to ensure a positive climate also when the problem is absent (or not detected). In the work of Saccardi et al. (2023), a first attempt to provide a similar climate was implemented using the E-Mate, a virtual agent that would comment on the responder ratings throughout the survey and react accordingly: when the ratings provided were mostly low scores, it would appear sad, and look happy when otherwise (examples can be seen in Figure 1). The E-Mate also presented a form of basic empathy, e.g. it would express regrets with low scores and happiness with positive scores. It is important to note that the researchers chose the sentences the E-Mate used, according to what sounded appropriate, without a rationale justifying the precise choice of statements. However, delivering the right support message is a complex challenge: the next section explains its implications.

### 2.5 Adapting support: personality and stressor

The regular use of a peer assessment tool, coupled with a virtual agent, represents an opportunity to detect group problems as they arise and deliver support to students simultaneously. However, knowing precisely what to say to make the respondent feel supported is not easy. Supporting attempts that are well-meant but wrongly phrased may cause undesired reactions, such as exacerbating negative emotions, inhibiting problem-solving, and increasing stress levels (Lehman and Hemphill, 1990; Dakof and Taylor, 1990; Davis et al., 1991; Barbee et al., 1998; Reynolds and Perrin, 2004). The definition of helpful support messages is, in itself, not universally established. In general, it is considered helpful to legitimize, recognize and elaborate on the recipients' feelings: this is defined as *person-centeredness* (Burleson, 2008); but this can be achieved in many different ways when phrasing a helpful message.

Previous research on the personalization of emotional support statements suggests that people adapt support messages choice and number to the recipient's personality and stressor - the stressful situation the recipient is facing (Smith et al., 2015; Dennis et al., 2013b,a; Kindness et al., 2017). For this reason, when designing a support agent, the recipient's situation (in the present research, group work) and their personality should both be taken into account. Personality can be defined as the unique set of qualities and dispositions that characterize each individual (Revelle, 1995; Goldberg, 1993). Among the numerous frameworks that can be used to describe personality, the Five Factor Model (FFM) (Goldberg, 1993; Rothmann and Coetzer, 2003) has been widely used in personalization research. In this framework, personality is mapped into five traits, each describing a stable pattern of thoughts and behaviors which determines one's response to the environment (Tellegen, 1991). This taxonomy will be used in the present work, with the following definitions:

1. **Extraversion**: the degree to which one is talkative, assertive, and energetic.
2. **Agreeableness**: the degree to which one is good-natured, cooperative, and trustful.
3. **Conscientiousness**: the degree to which one is orderly, responsible, and dependable.
4. **Emotional stability**: the degree to which one is calm, not neurotic, and imperturbable.
5. **Openness to experience**: the degree to which one is intellectual, imaginative, and independent-minded.

Among these personality traits, Conscientiousness has been positively associated with academic achievements, with students high in this trait reporting better academic performance (Hakimi et al., 2011; Imhof and Spaeth-Hilbert, 2013; Komarraju et al., 2009). This is not surprising, given that Conscientiousness has been linked to an individual's academic persistence and ability to organize information (Bratko et al., 2006). Students with high Conscientiousness are typically highly responsible and industrious; these characteristics guide them to strive for high academic achievements and to improve their performance (Hakimi et al., 2011; Bratko et al., 2006). Another trait closely related to students' academic behaviors is Emotional Stability: high levels of this trait are linked to better academic achievements (Chamorro-Premuzic and Furnham, 2003; O'Connor and Paunonen, 2007). A possible explanation for this finding is that students low in Emotional Stability are typically more anxious and more easily stressed: this heightened focus on their emotional state may divert attention away from academic tasks, which can further impair their academic performance (Poropat, 2009; De Raad and Schouwenburg, 1996; Chamorro-Premuzic and Furnham, 2014). In the work of Saccardi and Masthoff (2023), Emotional Stability was already considered a relevant factor for the adaptation of supportive messages following peer ratings on Productivity. The present work aims to extend these findings by including Conscientiousness, given the high relevance of the trait for academic achievements and the fact that it is connected to one's hardworking behavior, making it an obvious choice for Productivity. The findings about Conscientiousness will therefore be integrated with findings from related work on Emotional Stability as presented in Saccardi and Masthoff (2023).

### 2.6 Problem statement

This work extends related work by Saccardi et al. (2023) and Saccardi and Masthoff (2023) by studying how to adapt emotional support statements to a student working in a team. The design case is the survey by Saccardi et al. (2023), where a student rates another on five dimensions of teamwork, and a virtual agent provides feedback on the rating. Since the best emotional support depends on the recipient's personality, in this paper we study in particular how to adapt it to the rater's Conscientiousness level. As explained before, Conscientiousness is linked to being industrious and consequently closely related to Productivity, the teamwork attribute we will focus on.^1^ While all teamwork attributes are reported as relevant in the related work by Saccardi et al. (2023), we chose Productivity as it is related to a frequent issue in teamwork, namely that some students contribute a lot less than others, and in our own experience, when teachers only use one teamwork aspect in a peer-assessment on how well the team is going it tends to be this one. This is to some extent also shown by the results of Saccardi et al. (2023), where most students considered the attribute appropriate for a peer assessment survey and the attribute was used to discern between team members when filling it out.

An algorithm based on Productivity and Emotional Stability (another trait relevant for academic achievement) has already been proposed by Saccardi and Masthoff (2023); we will integrate their results on Emotional Stability with the results from the new study on Conscientiousness presented in this paper to create a more comprehensive algorithm that can adapt to both Conscientiousness and Emotional Stability [including the medium level of Emotional Stability that was not covered by Saccardi and Masthoff (2023)]. The choice to focus on Conscientiousness and Emotional Stability is further backed by related work that found that these are the only two Big 5 Personality traits that (1) clearly impact Productivity (Cubel et al., 2016)^2^ and (2) required adapting feedback on individual performance to Dennis et al. (2016).

## 3 Methods

This study builds on related work by Saccardi and Masthoff (2023), where a corpus of emotional support statements was collected, validated into emotional support categories, and then used to explore how a computer can adapt emotional support to a student with High or Low Emotional Stability rating another one on Productivity. In the present work, we extend this work by investigating how to adapt the same statements to a student with a High or Low Conscientiousness rating another one on Productivity.

### 3.1 Materials

#### 3.1.1 Categorized emotional support statements

**Figure 2:**
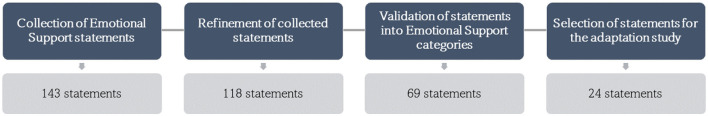
The process of statement collection, validation and selection from Saccardi and Masthoff (2023).

We used 24 emotional support statements from Saccardi and Masthoff (2023). These were part of a corpus produced in the related work by Saccardi and Masthoff (2023) in the following steps. First, an elicitation study was performed, where 23 university teachers wrote feedback to a student who had rated a teammate (with a score from 1 = awful to 5 = great) on one teamwork attribute [one from Quality of Contribution, Quality of Cooperation, Productivity, Reliability, and Friendliness, as proposed in the survey by Saccardi et al. (2023)]. They took the role of a teaching assistant and wrote as many alternative messages as they wished to the student who had done the rating. The resulting 143 statements were processed to remove duplicates, exclude inappropriate or too specific statements, rephrase questions into advice, and replace the student names with gender-neutral ones (Alex for the rater and Robin for the rated person): this resulted in 118 statements. Subsequently, in a validation study, participants categorized these statements into emotional support categories. The agreement between raters was measured by the *Free-Marginal Kappa* (κ) (Randolph, 2005), where 1 indicates complete agreement, 0.7 excellent and 0.4 moderate agreement. A statement was reliably categorized with κ ≥ 0.4. This resulted in 69 statements that were categorized into Celebration, Advice, Empathy, or Supported. The proposed categories were derived from the statements' content and related work (Dennis et al., 2013b,a; Smith et al., 2014; Dennis et al., 2016; Kindness et al., 2017) where they were used to provide emotional support to learners (on their own performance), informal carers, and community first-responders.^3^ In a second validation study, participants further categorized the validated Advice statements into sub-categories of Advice. This resulted in 10 Advice statements that were reliably categorized into Advice-Expectations (A-Exp), Advice-Feedback (A-Feed) and Advice-Improvement (A-Impr). Of these statements, 24 were selected to be used in the study by Saccardi and Masthoff (2023); the same statements were chosen to be used in the present study. A detailed description of the corpus creation process can be found in Saccardi and Masthoff (2023); a summary of the steps performed can also be seen in Figure 2; an overview of the emotional support categories can be found below and in Table 1.

- **Advice (A):** Statements in this category encourage Alex to take action to improve the situation (A-Impr), give feedback to Robin (A-Feed), or discuss their expectations (A-Exp). Providing advice in a supportive fashion is another important aspect of supportive communication, according to Burleson and Goldsmith (1996); Burleson (2003).
- **Celebration (C):** This category aims to express joy for Alex's positive experience. This category overlaps with the psychological concepts of *positive empathy* or *empathic joy*, which constitute the ability to share and celebrate others' positive emotions (Morelli et al., 2015; Smith et al., 1989).
- **Empathy (E):** In this context, empathy is defined as expressing regret and sympathy for the negative experience that Alex is feeling. The category of empathy here overlaps with the affective component of empathy, which focuses on how others' negative emotions affect oneself (Hoffman, 2001; Davis, 2018): the statements express that Alex's feelings are recognized and affect the sender of the supportive messages.
- **Supported (S):** Statements belonging to this category encourage involving the teaching staff in the situation, reminding Alex that the teachers are there to help if needed - as defined by Cobb (1976), social support also focuses on providing information that makes an individual believe that someone cares for them.

**Table 1:** Emotional support categories (Cat)^a^ from related work of Saccardi and Masthoff (2023).

**Cat**		**Definition**	**Example**
A	A-Exp	The statement suggests to Alex to clarify expectations with Robin.	*Make sure you make concrete agreements with one another*.
	A-Feed	The statement suggests to Alex to tell Robin their opinion/feelings on how things have gone.	*Robin may perform better if you gave them some feedback*.
	A-Impr	The statement suggests to Alex to discuss with Robin how Robin can improve in future.	*Perhaps you can talk with Robin on how to improve the quality of their work*.
C		The statement expresses joy for Alex's positive experience.	*Well done, keep on the good work*.
E		The statement expresses regret for Alex's negative experience.	*Sorry to hear Robin has not been very productive*.
S		The statement is about the teaching staff taking action.	*I will raise this with the teacher*.

a A = Advice; A-Exp = Advice-Expectations; A-Feed = Advice-Feedback; A-Impr = Advice-Improvement; C = Celebration; E = Empathy; S = Supported.

From the validated corpus of emotional support statements, 24 sentences (six for each emotional support category) applicable to Productivity were selected (for the Advice category, two statements per subcategory were chosen). The sentences and their category can be seen in Table 6.

#### 3.1.2 Personality stories

Two personality stories (Table 2) were adapted to be gender-neutral from the validated ones used in Dennis et al. (2016) to depict Alex as a High or Low Conscientiousness (Con) person. The stories used have been previously validated to describe one personality trait at a high or low level whilst depicting a neutral level on the other traits. The development and validation of the stories is described in Dennis et al. (2012); Smith et al. (2019). The stories were created using statements from a well-known validated FFM personality questionnaire (NEO-IPIP 20; Gow et al., 2005),^4^ and validated by participants rating the person depicted using another well-known FFM personality questionnaire (Mini-markers; Saucier, 1994).

**Table 2:** Stories used for High and Low conscientiousness (Con). Adapted from Dennis et al. (2016).

**Low Con**	**High Con**
Alex procrastinates and wastes their time. They find it difficult to get down to work. Alex does just enough work to get by and often doesn't see things through, leaving them unfinished. They shirk their duties and mess things up. Alex doesn't put their mind on the task at hand and needs a push to get started. Alex tends to enjoy talking with people.	Alex is always prepared. They get tasks done right away, paying attention to detail. Alex makes plans and sticks to them and carries them out. Alex completes tasks successfully, doing things according to a plan. They are exacting in their work; they finish what they start. Alex is quite a nice person, tends to enjoy talking with people, and quite likes exploring new ideas.

### 3.2 Procedure

A survey was created via Qualtrics and distributed via Prolific (prolific.co), a crowd-sourcing platform where participants complete online studies in exchange for a monetary reward (Peer et al., 2017). In a User-As-Wizard study (Masthoff, 2006), participants were first presented with a story describing the Conscientiousness of Alex, a fictional student (with one of the two stories presented in Table 2). They were then shown Alex's rating from 1 (awful) to 5 (great) of a teammate, Robin, on Productivity, defined as the quantity of work provided for the group project. Each rating was introduced as follows: “*Alex and Robin are two students working on a ten-week project together. After two weeks, Alex rated Robin on several aspects. On Productivity, Alex rated Robin 1 out of 5 (1=awful, 5=great)”*, with the given rating varying according to the condition. Participants were asked to take the part of the teaching assistant and select their feedback to Alex from the possible emotional support statements (the statements were presented in random order; they can be seen in Table 6). They could add multiple statements to the feedback if they wished to, and they could also provide comments. This resulted in a 2x5 between-subjects design, with two independent variables, namely Conscientiousness level (High and Low Con) and Productivity score (from 1 to 5), resulting in 10 different conditions. The dependent variable is the feedback participants produced, which we will consider on three levels of abstraction: (1) how many statements from each emotional support category participants used in their feedback, (2) for the Advice category how many statements from each Advice sub-category participants used, and (3) how often participants used specific statements (sentences) per (sub)category (from the statements shown in Table 6).

In this study, we did not specifically recruit teachers, as it would have been very hard to obtain the 300 teachers required (and another 300 for each study on the other personality traits and teamwork attributes we may need to conduct in the future). However, participants were constrained to build their feedback from validated statements that had originally been provided by teachers (as explained above). Additionally, participants brought with them their own experiences of working in teams, given how universal teamwork is in education and beyond, and should therefore have been capable of judging the appropriateness of statements from a recipient's point of view. We note that related work on emotional support (e.g. Dennis et al., 2016) has taken a similar approach.

## 4 Results

### 4.1 General overview

300 participants (150 male and 150 female) participated in the experiment, 30 per condition, aged 19–70 (*M* = 29.17, *SD* = 9.77). A 2-way MANOVA was performed to test the effect of Conscientiousness level (High or Low Con) and Productivity score (from 1 to 5) on the emotional support category (Advice, Celebration, Empathy, Supported). Main effect analysis showed a significant effect of Productivity score on the multivariate pattern of all individual categories of emotional support [F_(16, 877.44)_=12.154, *p* < 0.001]).^5^ Furthermore, an interaction between Productivity score and Con level was found for the Celebration category [F_(4, 290)_ = 3.712, *p* = 0.006].

**Figure 3:**
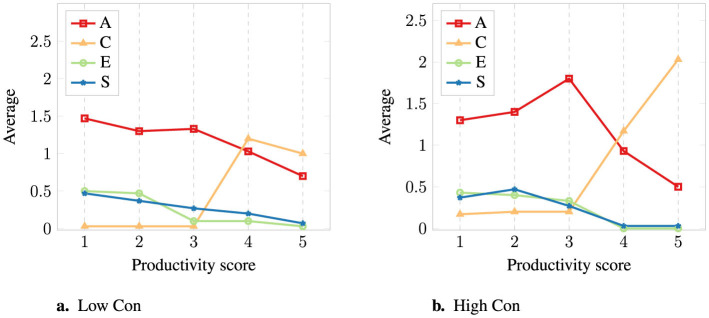
Average number of statements used from each category for each productivity score and **(a)** Low or **(b)** High Con.

**Figure 4:**
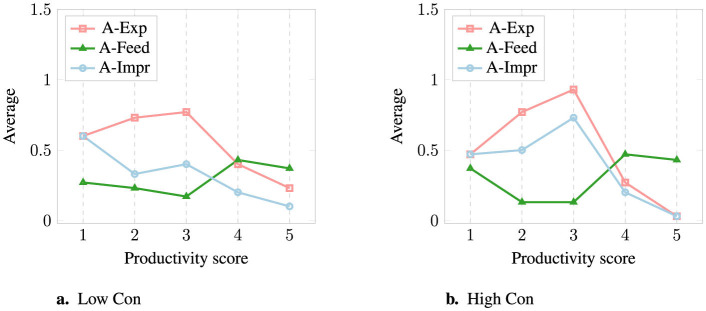
Average number of statements used from each Advice subcategory for each Productivity score, for **(a)** Low and **(b)** High Con.

An overview of the average amount of statements used for each category and condition can be seen in Figure 3 and in Table 3 including counts, medians, and standard deviations (A, Advice; C, Celebration; E, Empathy; S, Supported).^6^ For the category Advice, an overview of the averages for subcategory and condition can be seen in Figure 4 and in Table 4 with counts, medians, and standard deviations (A-Exp, Advice-Expectations; A-Feed, Advice-Feedback; A-Impr, Advice-Improvement).

**Table 3:** Overview of emotional support categories used for each Productivity score (Sc) and Con level. For each emotional support category the total number of statements used in each condition (N), the average number (Avg), the median (M) and the standard deviation (SD) are reported. The last column (M-Dec) summarizes the decisions based on the Median.

		**A**				**C**				**E**				**S**				**M-Dec**
**Sc**	**Con**	**N**	**Avg**	**M**	**SD**	**N**	**Avg**	**M**	**SD**	**N**	**Avg**	**M**	**SD**	**N**	**Avg**	**M**	**SD**	
1	Low	44	1,47	1	1,57	1	0,03	0	0,18	15	0,50	0	0,57	14	0,47	0	0,57	A
	High	39	1,30	1	1,02	5	0,17	0	0,38	13	0,43	0	0,50	11	0,37	0	0,56	A
2	Low	39	1,30	1	0,75	1	0,03	0	0,18	14	0,47	0	0,78	11	0,37	0	0,61	A
	High	42	1,40	1	0,93	6	0,20	0	0,48	12	0,40	0	0,56	14	0,47	0	0,82	A
3	Low	40	1,33	1	0,84	1	0,03	0	0,18	3	0,10	0	0,31	8	0,27	0	0,64	A
	High	54	1,80	1,5	1,27	6	0,20	0	0,61	10	0,33	0	0,76	8	0,27	0	0,58	2A
4	Low	31	1,03	1	0,85	36	1,20	1	1,54	3	0,10	0	0,31	6	0,20	0	0,48	C,A
	High	28	0,93	1	0,78	35	1,17	1	1,02	0	0,00	0	0,00	1	0,03	0	0,18	C,A
5	Low	21	0,70	1	0,65	30	1,00	1	0,98	1	0,03	0	0,18	2	0,07	0	0,25	C,A
	High	15	0,50	0	0,57	61	2,03	2	1,40	0	0,00	0	0,00	1	0,03	0	0,18	2C

**Table 4:** Overview of the advice subcategory usage for each Productivity score (Sc) and Con level. For each subcategory, the total number of statements used in each condition (N), the average number (Avg), the median (M) and the standard deviation (SD) are reported. The last column (A-Dec) summarizes the decisions.

		**A-Exp**				**A-Feed**				**A-Impr**				
**Sc**	**Con**	**N**	**Avg**	**M**	**SD**	**N**	**Avg**	**M**	**SD**	**N**	**Avg**	**M**	**SD**	**A-Dec**
1	Low	18	0,60	0	0,77	8	0,27	0	0,52	18	0,60	0,5	0,67	A-Impr
	High	14	0,47	0	0,68	11	0,37	0	0,56	14	0,47	0	0,57	A-Impr
2	Low	22	0,73	1	0,64	7	0,23	0	0,43	10	0,33	0	0,48	A-Exp
	High	23	0,77	1	0,50	4	0,13	0	0,35	15	0,50	0	0,57	A-Exp
3	Low	23	0,77	1	0,57	5	0,17	0	0,38	12	0,40	0	0,56	A-Exp
	High	28	0,93	1	0,83	4	0,13	0	0,35	22	0,73	1	0,69	A-Impr, A-Exp
4	Low	12	0,40	0	0,50	13	0,43	0	0,50	6	0,20	0	0,41	A-Feed
	High	8	0,27	0	0,52	14	0,47	0	0,51	6	0,20	0	0,41	A-Feed
5	Low	7	0,23	0	0,57	11	0,37	0	0,49	3	0,10	0	0,31	A-Feed
	High	1	0,03	0	0,18	13	0,43	0	0,50	1	0,03	0	0,18	A-Feed

A post hoc Tukey HSD pairwise comparison was also conducted; the resulting homogeneous subsets of scores on the number of statements for each category and Con level can be seen in Table 5. Overall, we observe the following trends:

- **Scores 1 and 2**. For both High and Low Con, people provided Advice: they mostly suggested discussing how to improve the situation for score 1 and discussing expectations for score 2. The advice does not differ according to the Con level, although some people reported a difference in the comments; for example, one participant noted for the High Con condition: “*Robin might be unaware of the lack of productivity; however, it could be Alex's meticulous attention to detail that makes Robin look like [they] are not productive”*.
- **Score 3**. For Low Con, people once again suggested discussing expectations. This is not surprising, given that Alex's expectations not only deeply influence the evaluation of a middle score, but are also not obvious based on the personality story. A person who tends to procrastinate may not expect much from a teammate: as one participant commented, “*Alex cannot expect Robin to be more productive than he is”*. It is also possible that Alex's score is not reflecting reality: one participant reported, “*I would offer these comments to them but I would suspect that Robin is pulling the weight of the work. Or no work is getting done as neither is very productive”*. In both cases, clarifying expectations seems a good idea. For High Con, participants recommended discussing expectations but also improvement. We observe here a first difference between Con levels. A possible interpretation is that Alex is presented as a precise, exact person, and as such, it may be crucial to work on improving the situation, as noted by one participant: “*Robin might not be a perfectionist like Alex is”*.
- **Score 4**. For both High and Low Con, participants recommend providing feedback to Robin and congratulating Alex. It is important to note that, as can be seen in Table 6, the preferred A-Feed statement used is “*I recommend you tell Robin you are quite happy with their productivity. They will be happy to hear so.”*, which is in line with 4 out of 5 being a relatively high score. The criteria for the recommendation decision do not result in any difference between High and Low Con; it is interesting to note, however, a difference on a descriptive level while looking at the averages of subcategories in Table 4. While providing feedback remains the preferred option, many also recommend discussing expectations in the Low Con case. A possible interpretation is that Alex is presented as a procrastinating person, and people doubt that the score is truthful or still suspect that Robin is working more than Alex, as can be derived by participants' comments: “*Is Robin being productive for both of them - it's a team assignment and both need to pull their weight equally”*.
- **Score 5**. People recommend once again giving positive feedback to Robin and congratulating Alex. We note here another difference between High and Low Con: when Alex is presented as a High Con person, more celebration is suggested, as showed by the recommendation of two statements instead of one (and similarly giving positive feedback when looking at the homogeneous subset in Table 5). Possibly, since Alex is presented as a precise, planning person, a 5 out of 5 is especially positive - Robin, as a teammate, was scored the highest possible by someone scrupulous and possibly demanding and this therefore requires extra celebration.

**Table 5:** Homogeneous subsets of score per Con level and score for each emotional support category (Cat). For each subset, the average is reported (SubAvg), together with the decisions made based on subsets and medians.

				**Subset decision**					**Median decision**				
**Con**	**Cat**	**Subset**	**SubAvg**	**1**	**2**	**3**	**4**	**5**	**1**	**2**	**3**	**4**	**5**
Low	A	1,2,3,4	1,28	A	A	A	A	A	A	A	A	A	A
		2,3,4,5	1,09										
	C	1,2,3	0,03	-	-	-	C	C	-	-	-	C	C
		4,5	1,10										
	E	1,2	0,48	-	-	-	-	-	-	-	-	-	-
		3,4,5	0,08										
	S	1,2,3,4	0,33	-	-	-	-	-	-	-	-	-	-
		2,3,4,5	0,23										
High	A	1,2,3	1,50	A	A	2A	A	A	A	A	2A	A	-
		1,2,4	1,21										
		4,5	0,72										
	C	1,2,3	0,19	-	-	-	C	2C	-	-	-	C	2C
		4	1,17										
		5	2,03										
	E	1,2,3	0,39	-	-	-	-	-	-	-	-	-	-
		3,4,5	0,11										
	S	1,2,3	0,37	-	-	-	-	-	-	-	-	-	-
		1,3,4,5	0,18										

**Table 6:** Statements used for each emotional support category. For each statement, the number of occurrences in participants' feedback per condition is reported when relevant to the algorithm. The *Free-Marginal kappas* from the validation of statements into support categories and Advice subcategories are reported (*k*).

					**1**		**2**		**3**		**4**		**5**	
**Cat**		**#**	***k***	**Statement**	**L**	**H**	**L**	**H**	**L**	**H**	**L**	**H**	**L**	**H**
**A**	Exp	1	0.56	If you have not yet done so, agree clear expectations with Robin on productivity.			5	8	4	10				
		2	0.57	I think you need to discuss with Robin the types of expectations you both have on productivity, and come to some agreement.			17	15	19	18				
	Feed	1	0.44	Tell Robin how you feel about them not having been so productive.							1	1	1	0
		2	0.71	I recommend you tell Robin you are quite happy with their productivity. They will be happy to hear so.							12	13	10	13
	Impr	1	0.61	Perhaps you can talk with Robin on how to improve their productivity.	10	5				8				
		2	0.56	Perhaps you can talk with Robin on how to improve their productivity even more.	8	9				14				
C		**1**	0.73	Delighted that you are so happy with Robin's productivity.							8	5	10	7
		2	0.83	Good to see the collaboration is going well!							7	8	6	17
		3	0.83	Delighted to hear this.							3	2	3	6
		4	0.94	Congratulations.							1	3	2	3
		5	0.64	Well done, keep up the good work.							4	8	4	18
		6	0.94	Congratulations on having a productive teammate.							13	9	5	10
E		1	0.84	I'm sorry you are having some difficulties.										
		2	0.84	I'm sorry you are having a tough time.										
		3	0.64	Really sorry to hear that Robin's productivity did not meet your standards.										
		4	0.65	Really sorry that Robin is not pulling their weight.										
		5	0.79	Really sorry to hear.										
		6	0.69	Sorry that Robin did not do so much.										
S		1	0.68	The teacher will talk to Robin.										
		2	0.69	Please let me know if you would like me to raise this with the teacher.										
		3	0.73	I will tell the teacher.										
		4	0.74	I will raise this with the teacher.										
		5	0.74	I will let the teacher know so that they can help you.										
		6	0.47	Please let me know if you would like the teacher to talk with Robin.										

The gray share indicates cells where the count is not relevant.

### 4.2 Selection of statements

The analysis resulted in a series of decisions indicating which emotional support category to use for each Con level and Productivity score (Sc). This section describes every step of these decisions.

#### 4.2.1 Selection of categories

For each combination of the Con level and Productivity score, the decisions regarding which emotional support category or subcategory to select for the emotional support algorithm were made in two different ways:

1. Based on the Medians (*M*) reported in Table 3. Decisions are shown in Table 3, column “M-Dec”, and repeated in Table 5, column “Median decision”, to allow for comparison with the Subset decisions:
  - When the median was *M* < 0.5, no statements of that category were included.
  - When the median was 0.5 ≤ *M* < 1.5, one statement was included.
  - When the median was 1.5 ≤ *M*, two statements were included.
2. Based on homogeneous subsets average (*SubAvg*) as shown in Table 5, where decisions are provided in column “Subset decision”:
  - When the subset average was *SubAvg* < 0.5, no statements of that category were included.
  - When the subset average was 1 ≤ *SubAvg* < 1.5, one statement was included.
  - When the subset average was 1.5 ≤ *SubAvg*, two statements were included.
  - When a score is part of multiple subsets that would lead to different decisions, then the average was calculated for the combination of those subsets, and the decision based on that.

Table 5 shows that the decisions based on homogeneous subsets and medians are almost identical, so the methods provide corroboration of each other (we will return to the one case in which they differ in Section 5).

#### 4.2.2 Selection of Advice subcategories

Whenever a statement from the Advice category was recommended, the subcategories of Advice (overview in Table 4, Figure 4) were chosen in the following way:

- When the Median of the Advice subcategory was *M* = 0.5 or *M* = 1, a statement was included.
- When the Median was *M* = 0, but a statement had to be included, the subcategory with the highest average (Avg) was chosen. This was the case for Score 4 and score 5.
- For Score 1 and High Con, the averages of A-Exp and A-Impr were the same; A-Impr was chosen to mimic the decision for Low Conscientiousness.

#### 4.2.3 Order of statement categories

When multiple categories or subcategories were chosen, it was necessary also to decide on the order in which to provide them. To do so, we considered the order participants used when they used both:

1. Score 3, High Con: when A-Exp and A-Impr were used together, in 50% of cases people started with A-Exp and in 50% with A-Impr. We opted for A-Impr followed by A-Exp to be in line with results in Saccardi and Masthoff (2023); when the two subcategories are used together in their algorithm, it is always in the order A-Impr followed by A-Exp. Consistency across algorithms facilitates the integration of the two algorithms later in this paper.
2. Score 4: A and C were suggested for score 4. For both Low and High Con, C was provided first (in 78% of the cases for Low Con, and 79% of the cases for High Con); therefore, we decided on C-A.
3. Score 5: in High and Low Con, both A and C were suggested in the subset decision. In the majority of cases (65%), C was used first. Therefore, we decided on C-A.
4. Score 5, High Con: two statements from the C category were suggested; looking at Table 6, the most used candidates are C2 and C5. C2 was used first in most cases (55%); therefore, we decided on C2-C5.

#### 4.2.4 Selection of individual statements

Each emotional support category consisted of six statements, with two statements for each subcategory of Advice. Table 6 shows how often each statement was used for each combination of score and Con level when relevant to the algorithm: to decide the precise statement to use, we looked at the one used most often for each case.

## 5 Algorithm creation

### 5.1 Step 1: initial algorithm creation

The decisions in Table 5 were used to produce an initial algorithm (Algorithm 1), which corresponds to Table 7 column “Conscientiousness”. In the case of Score 5 for High Con, the decisions based on the median and the subset were not in line, with the median suggesting no Advice (*M* = 0) and the subset average suggesting one (*SubAvg* = 0.72). We opted to add one advice statement to make the decision consistent with what was chosen for Low Con.

**Algorithm 1:**
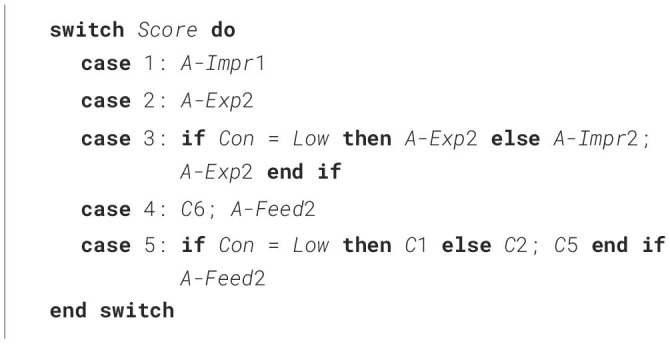
Adaptation to score and Conscientiousness (High and Low).

**Table 7:** Decisions in our study on Conscientiousness (reflected in Algorithm 1) and in related work (Saccardi and Masthoff, 2023) on Emotional Stability for each Productivity score (Sc), and decisions based on this for Medium Conscientiousness (Dec Medium Con) (reflected in Algorithm 2).

**Sc**	**Conscientiousness**		**Emotional stability**		**Decisions**
	**Low**	**High**	**Low**	**High**	**Medium Con**
1	A-Impr1		A-Exp2	A-Impr1; A-Exp2; E1	A-Impr1; A-Exp2
2	A-Exp2		A-Exp2; E3	A-Impr1; A-Exp2; E3	A-Exp2; E3
3	A-Exp2	A-Impr2; A-Exp2	A-Exp2		A-Exp2
4	C6; A-Feed2		C1; A-Feed2	C2; C5; A-Feed2	C6; A-Feed2
5	C1; A-Feed2	C2; C5; A-Feed2	C1; C2; A-Feed2		C1; C2; A-Feed2

### 5.2 Step 2: decisions on Medium Conscientiousness

Algorithm 1 considered score and two Conscientiousness levels, namely High or Low. Here, we extend it by including Medium Conscientiousness, leading to the decisions shown in Table 7 (column “Dec Medium Con”) and Algorithm 2.

**Algorithm 2:**
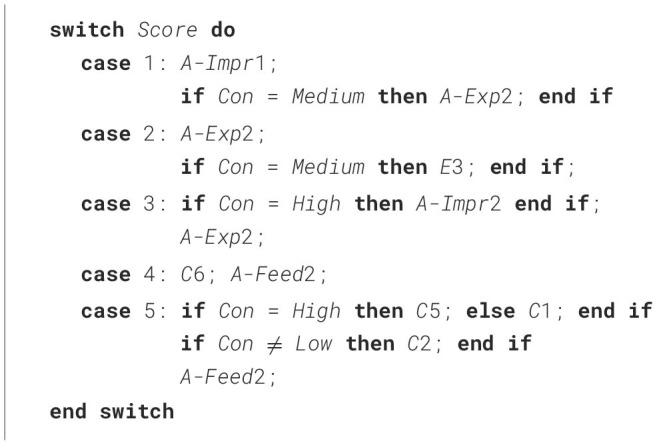
Adaptation to score and Conscientiousness (Medium level included).

Our study only investigated High and Low Conscientiousness and not Medium Conscientiousness, as the validated personality stories used only exist for high and low trait levels. The related work's study Saccardi and Masthoff (2023) used a similar approach to create an algorithm adapting support statements to High and Low Emotional Stability (ES), resulting in the decisions shown in Table 7, column “Emotional Stability”. To create the algorithm, they used validated personality stories depicting High and Low Emotional Stability (from Smith et al., 2019; Dennis et al., 2012), and asked participants to provide appropriate feedback for each combination of a Productivity score (from 1 to 5) and Emotional Stability level (High and Low), in a similar fashion to the present work. These personality stories were validated (as reported in Smith et al., 2019; Dennis et al., 2012) to depict *a neutral level on the other traits* whilst correctly depicting a high or low trait level. So, the related work's study Saccardi and Masthoff (2023) on the impact of Emotional Stability (ES) depicted neutral Conscientiousness, and its results may therefore help decide what to do for Medium Conscientiousness (Medium Con).

Table 7 summarizes the decisions per score and personality trait level for our study and the related work (Saccardi and Masthoff, 2023),^7^ and the decisions we are making here for Medium Conscientiousness.

We base the Medium Conscientiousness decisions on the following applied in that order:

1. When a statement is used for both High and Low Con, and used for either High or Low ES, we use it for Medium Con. This occurs for A-Impr1 for score 1, A-Exp2 for scores 2 and 3, and A-Feed2 for scores 4 and 5.
2. When a statement is used for both High and Low ES, and used for either High or Low Con, we use it for Medium Con. This occurs for C1 and C2 for score 5.^8^
3. When a statement category is used for both High and Low Con, and used for either High or Low ES, we will use it for Medium Con, choosing the statement that is most commonly used. This occurs for category C in score 4, where we opt to use C6 as it is used most often.^9^
4. When a statement is used for both High and Low ES, and not used for either Con condition, we will use it for Medium Con, assuming it can be made plausible why participants opted against its use for High and Low Con. This occurs for A-Exp2 for score 1, and E3 for score 2. We will discuss these cases below.
5. When a statement category is used for only one of the two Con conditions and not used for either of the ES conditions, then it will not be used for Medium Con. This occurs for A-Impr (statement A-Impr2) for score 3.
6. When a statement category is used for only one of the two ES conditions and not used for either of the Con conditions, then it is not used for Medium Con. This occurs for Empathy (statement E1) for score 1 and A-Impr (statement A-Impr1) for score 2.

The resulting decisions can be seen in Table 7. Combining these decisions with Algorithm 1 results in Algorithm 2, which corresponds to Table 7 columns “Conscientiousness” and “Dec Median Con” combined.

When making the decisions, there were two noteworthy cases. The first noteworthy case is the inclusion of A-Exp2 (“*I think you need to discuss with Robin the types of expectations you both have on productivity, and come to some agreement.”*) for Medium Con but not High or Low Con for score 1. A possible explanation is that participants assumed that the High Cons Alex's standard for Productivity would be very high, and much too far apart from the 1 rating they had given Robin to sensibly discuss common expectations. For the Low Con Alex, we have many comments to indicate that participants saw them as part of the problem (e.g. “*Sometimes it's easier to see the flaws in others rather than ourselves”;“Alex made Robin lazy”*), and may therefore have been more hesitant to suggest agreeing on common expectations. The second noteworthy case is the inclusion of E3 (“*Really sorry to hear that Robin's productivity did not meet your standards”*) for Medium Con but not High or Low Con for score 2. Again, it is possible people felt the High Con Alex may have had standards that were too high (“*Robin might be unaware of the lack of productivity; however, it could be Alex's meticulous attention to detail that makes Robin look like [they] are not productive.”*), and that the Low Con Alex may have not been very productive themselves, making Empathy less likely.

### 5.3 Step 3: Including Emotional Stability

Algorithm 2 considered score and Conscientiousness level (High, Low and Medium). Here, we integrate the related work's results (Saccardi and Masthoff, 2023) regarding Emotional Stability (ES) into the algorithm, leading to the decisions shown in Table 8 and Algorithm 3.

**Table 8:** Decisions based on step 3 for each case of Productivity score (Sc), Emotional Stability level, and Conscientiousness level. These decisions are reflected in Algorithm 3.

**Sc**	**Emotional stability**	**Conscientiousness**	**Decisions**
1	High	High, Low	A-Impr1
		Medium	A-Impr1; A-Exp2; E1
	Medium	High, Low	A-Impr1
		Medium	A-Impr1; A-Exp2
	Low	Any	A-Exp2
2	High	High, Low	A-Impr1; A-Exp2
		Medium	A-Impr1; A-Exp2; E3
	Medium, Low	Medium	A-Exp2; E3
3	Any	High	A-Impr2; A-Exp2
		Medium, Low	A-Exp2
4	High, Medium	Any	C6; A-Feed2
	Low		C1; A-Feed2
5	Any	High	C5; C2; A-Feed2
		Medium	C1; C2; A-Feed2
		Low	C1; A-Feed2

**Algorithm 3:**
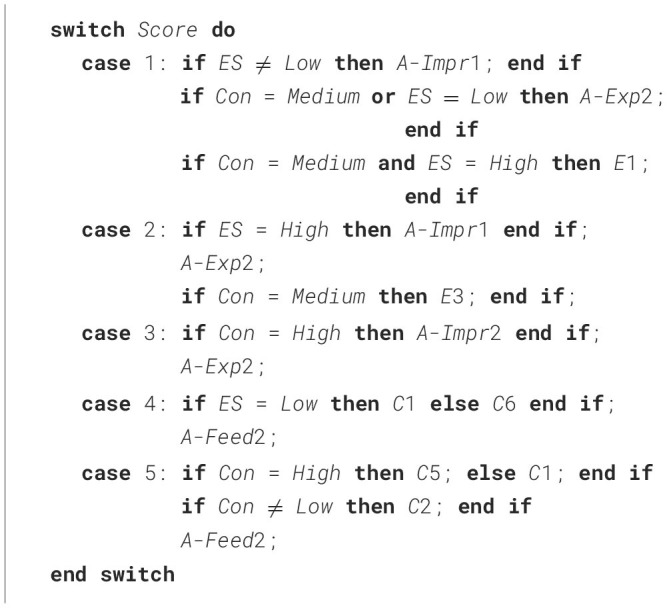
Adaptation to score, Conscientiousness and Emotional Stability.

Decisions were made as follows:

- For scores 3 and 5, the emotional support provided in Algorithm 2 is identical to that provided for both Low and High ES (see Table 7), so no change is needed.
- For score 2, the emotional support provided in Algorithm 2 is identical to that provided for Low ES. For High ES, based on Table 7, an additional A-Impr1 (“*Perhaps you can talk with Robin on how to improve their productivity”*) is added to the new algorithm. It makes sense that this is only done for High ES, as it may be quite scary to have such a conversation for a more nervous person.
- For score 1, the emotional support provided in Algorithm 2 differs in two places from the ES decisions in Table 7 from Saccardi and Masthoff (2023). Firstly, for Low ES, A-Impr should not be used (for similar reasons as discussed above), hence, we will use A-Exp instead in line with Saccardi and Masthoff (2023) for this case. Secondly, for High ES, E1 (“*I am sorry you are having some difficulties”*) is used. We decided to add E1 only for Medium Con, given the discussion in one of the noteworthy cases in Step 2 regarding the lack of Empathy shown for High and Low Con. The algorithm is modified accordingly.
- For Low ES for score 4, the emotional support provided in Algorithm 2 differs only in the specific C statement used; Low ES used C1 (“*Delighted that you are so happy with Robin's productivity”*) compared to the algorithm using C6 (“*Congratulations on having a productive teammate”*). Considering the details in Saccardi and Masthoff (2023), there was indeed much more support for C1 for Low ES than for C6, so we adapted the new algorithm accordingly. Maybe participants felt that C6 may to a neurotic (Low ES) person give the impression that you were less impressed with their own productivity or they felt that C1 sounded more positive than C6 and that the Low ES person could do with cheering up. In contrast, C6 was more popular for High and Low Con. Maybe participants felt that a score of 4 for a High Con person would not be a cause for too much happiness, as they may have their standards higher, and too much celebration would also not be appropriate for a Low Con person given they may have assumed that person may have been less productive themselves.
- For High ES for score 4, the main difference is that the emotional support provided in Algorithm 2 contained only one C statement compared to the two C statements recommended. As already argued in a footnote before, the choice for C2 rather than C6 in Saccardi and Masthoff (2023) was arbitrary, so the difference is mainly in whether the second C (namely C5) should be added. We decided against adding this for two reasons. Firstly, the addition of the second C was controversial even in Saccardi and Masthoff (2023), as their analysis based on the mediums argued for only one C (compared to the subset analysis which led to the 2 Cs). Secondly, we find it hard to provide a reason for providing an emotionally stable person with more celebratory messages. So, the algorithm was not changed for High ES for score 4.

## 6 Discussion

In this paper, we first investigated how people adapt the selection of emotional support statements to a student (Alex) rating another team member (Robin) based on the rater's Conscientiousness level and the score assigned to the teammate's Productivity. The results provide multiple insights into people's choices of emotional support and possible applications of the resulting algorithm.

**People adapt emotional support to personality and situation**. The findings corroborate previous research showing that people adapt emotional support statements to personality and stressors (Smith et al., 2015; Dennis et al., 2013b; Saccardi and Masthoff, 2023). People adapt emotional support to the rating Alex assigned to their teammate Robin, and to Alex's level of Conscientiousness. When scores are low, 1 and 2, they recommend giving advice, suggesting that Alex discusses with Robin how to improve and the types of expectations they both have on Productivity respectively. When the score is 3, they suggest talking about expectations, and when Alex is presented as a highly conscientious person, they recommend also discussing improvements. When scores are high, 4 or 5, people recommend congratulating and giving positive feedback to the teammate - there is reason to celebrate especially when Alex is presented as a highly conscientious person, as shown by two celebration statements for that condition.

**An algorithm that substantially expands related work**. The decisions resulted in an algorithm showing how to adapt emotional support to High or Low Conscientiousness levels and Productivity score, shown in Algorithm 1. This work was then extended by adding decisions for Medium Conscientiousness level, shown in Algorithm 2, and including the related works' results on Emotional Stability, leading to the creation of Algorithm 3. By integrating our results with the related work by Saccardi and Masthoff (2023), we obtained an algorithm that can adapt emotional support based on the rater's Emotional Stability, Conscientiousness, and given score on Productivity (a frequently used dimension to assess teamwork contributions). We focused on Conscientiousness and Emotional Stability because of their high relevance to academic achievement and productivity. This algorithm has value in its own right, as it can be used already to improve the feedback in the peer assessment survey in Saccardi et al. (2023) for Productivity or to provide feedback in a peer assessment that only focuses on Productivity.^10^ Additionally, the algorithm provides an important basis for extension to one that can tackle ratings on multiple teamwork aspects and personality traits.

**Practical outcome of the findings**. This study is based on the design case of Saccardi et al. (2023), where a student working in a group fills out a peer assessment survey, rating their teammates on different dimensions of teamwork, and a virtual agent provides emotional support statements based on the ratings. Such a peer assessment survey offers the possibility of reaching out individually to students working in groups, monitoring their perspective of how the group work is going, and supporting them with personalized feedback based on their ratings. However, in the work conducted by Saccardi et al. (2023), the feedback provided by the virtual agent is arbitrary, while it is necessary to consider the feedback the students receive carefully. The present study, combined with the results from the related work Saccardi and Masthoff (2023), presents an important step toward building a reliable framework of support statements to be used by a virtual agent in designs such as Saccardi et al. (2023), able to provide emotional support based on all Big 5 personality traits and all aspects of teamwork (five according to Saccardi et al., 2023). The benefits of a new channel of support toward students are multiple: it allows for a virtual coach to reach out individually to every student working in a team, collecting their experiences, and providing personalized feedback based on their unique viewpoint of the group work. It enables teachers to promote a positive climate toward group work through a digital medium - even when the scores provided are low and possibly signal dissatisfaction in the group work. It ensures that every single student is attended to at least by a virtual “mediator” - whose verbal feedback has been built from teachers' input in the first place, selected by people to be appropriate for the student's personality and scores given. Lastly, it does not constitute an additional effort to search for support, but it is embedded in a survey that students fill out as part of their course, ensuring that also the ones who are not actively looking for help still feel seen and supported. Whilst the example in this paper is that of students working in a team, applicability extends beyond computer-supported-collaborative-learning to teamwork more general.

## 7 Future directions

### 7.1 Extensions to the algorithm that provides feedback to the rater

*Extension to other personality traits*. Whilst this paper focused on Conscientiousness and Emotional Stability for good reasons (see above), the impact of other personality traits could also be investigated. For example, a rater's Agreeableness may impact their ratings of others, and this may need to be considered in the feedback provided.

*Extension to other teamwork attributes*. Whilst this paper focused on Productivity for a good reason and the algorithm is valuable in its own right (see above), considering the use case of Saccardi et al. (2023), we will extend the research to other teamwork aspects, using a similar methodology. The aim will be to create an algorithm a virtual agent can use throughout the peer assessment survey of Saccardi et al. (2023), covering all cases where feedback needs to be provided. Based on the relative importance participants in Saccardi et al. (2023) attached to teamwork attributes, we will study Quality of Contribution next: initial work can be seen in Saccardi and Masthoff (2024). The extension to other attributes poses, however, some challenges such as how to combine different scores on multiple attributes. For example, if a teammate is highly productive, the algorithm currently congratulates the rater for having such a teammate. However, what to do if on the next peer assessment question this teammate turns out to have produced low-quality work? To mimic a conversational style, it may be best to provide feedback after each question, but then the feedback already provided on Productivity may need to be considered when commenting on Quality of Contribution.

*Extension to multiple peer assessments over time*. As the design case is based on a peer assessment delivered multiple times during a team project, we also will consider how to adapt feedback to multiple assessments over time. For example, when there are improvements or new issues arise. This requires investigations into how to change emotional support when an issue is repeated, or getting worse or better. For example, a suggestion to align expectations may be less appropriate if this was suggested already the first time an issue appeared. More user studies are needed for this. Validation of a larger set of emotional support message phrasings is also needed so that feedback over time is not too repetitive.

*Extension to multiple teammates*. This paper studied the provision of feedback to a person who rated one teammate. However, the peer assessment survey has already been used for larger teams, with the rater having rated multiple teammates Saccardi et al. (2023). Whilst it is possible to use the algorithm also in such a case, providing feedback related to each teammate in turn, we want to investigate also how this can be made more sophisticated. This may for example include (1) adapting the feedback about later-rated teammates to the feedback already provided, (2) combining the feedback for multiple teammates, or (3) adding feedback about all teammates to feedback per teammate.

### 7.2 Providing feedback to others

This article focused on providing feedback to the person who rated their teammates. We also started working on providing feedback to others.

*Providing feedback to the person rated*. This is challenging when the person has been rated by multiple teammates. The main issue is how to combine ratings, particularly when contradictory. This could also include the provision of feedback on how an individual compares to others and feedback to the group as a whole. Initial work on this, discussing the many issues involved and several proposed formalisations and ways of tackling this, is provided in Masthoff and Saccardi (2024).

*Providing feedback to teachers on the wellbeing of groups*. Teachers could be provided with a summary of how all groups are doing, highlighting those that are experiencing serious problems and may need a teacher intervention in addition to the automatic feedback already provided. This requires combining issues with individual team members into a severity for the group as a whole. Initial work is in Masthoff and Saccardi (2024); the results from initial user studies on this issue still need to be reported.

### 7.3 Evaluating the algorithm: opportunities and challenges

The algorithm in this paper was derived from the data from two large-scale studies with 500 participants in total (one in this paper and one in Saccardi and Masthoff, 2023), using statements that had been provided by 23 teachers and that were validated in another study. The algorithm was checked by the teachers who authored this paper before it partially was already applied in a peer assessment in a course.^11^ Additionally, we want to evaluate the impact of the resulting algorithm.

To evaluate the impact, students should judge the provided emotional support's appropriateness and whether it contributes to their feeling supported throughout their group work project. This can be done in a field study in a course by implementing our algorithm in the peer assessment survey from related work Saccardi et al. (2023). Students can then indicate to which degree they felt supported, compared to a system without the algorithm. An initial evaluation of a peer assessment survey with a virtual agent providing feedback was already done in Saccardi et al. (2023), who reported a positive reception of the survey overall. However, the emotional support provided by the virtual agent in that evaluation was rather arbitrary; the algorithm from this paper can be used to improve this.

Evaluating such an adaptive emotional support algorithm presents many challenges:

- **Participant sample**. In related work Saccardi et al. (2023), the number of students filling out the survey was too limited to test the algorithm's efficacy reliably given the very many conditions that need to be tested. Whilst it is possible to make it compulsory for students to fill out such a survey for class purposes, it is not ethical to force students to take part in research.
- **Variables distribution**. Testing in a course entails no control over the presence or absence of group issues, making testing all relevant cases especially challenging. A similar obstacle concerns the distribution of students' personalities, which can be diverse and does not guarantee to cover all levels of each personality trait, certainly not in combination with particular group issues.
- **Ethical concerns**. An obvious choice for testing the algorithm would be to compare the emotional support experienced by a sample receiving support to that of a sample without it. Unfortunately, the lack of support^12^ for half the population sample creates significant ethical issues when done in a classroom setting, rendering this methodology unusable and making the quest for an appropriate experimental design particularly challenging.
- **Confounding variables**. Expressing interest toward one's well-being may represent a type of support on its own, as it represents interest and care toward one's emotional status (Burleson and Goldsmith, 1996; Thoits, 1995). This may bias the evaluation results (as found by Nguyen and Masthoff, 2009). Unfortunately, asking people about how they feel before and after the survey (or in any other form of control condition) cannot be avoided. Additionally, group work scenarios vary widely and are often quite complex. This complexity makes it hard to predict which other factors may affect the algorithm's effectiveness, limiting the generalizability of evaluation results.

A way to partially circumnavigate the mentioned evaluation challenges is implementing the survey in a different, controlled environment. In this line of research, we are currently investigating the implementation of a similar peer assessment survey in a game environment where part of the team is composed of AI players without the knowledge of the other participants. This environment allows for manipulation of the issues a group encounters through the addition of, for example, a slacking AI teammate, or one that actively hinders others' contribution, and so on. The online deployment of such a survey may also enable us to recruit a wider variety of personality trait levels, which allows for more comprehensive testing of possible cases.

## Data Availability

The raw data supporting the conclusions of this article will be made available by the authors, without undue reservation.

## Footnotes

^1^We focused on just one attribute, as (1) the chance of finding spurious effects (Type I statistical error) would have been too high otherwise (or with Bonferroni corrections, the tests would have lacked statistical power), (2) with a within-subject design, the burden on participants would have been too large and interaction effects may have occurred (see Future work), (3) with a between-subject design, the number of participants required would have been too large.

^2^And may therefore impact the rater's view on somebody else's Productivity when comparing it to their own.

^3^In addition to the categories presented here, Emotional Reflection and Reassurance were used in the validation study, but were removed as they did not result in enough validated statements.

^4^Sometimes adding a statement to ensure a neutral depiction of the other traits, for example, “Alex tends to enjoy talking with people” was added to ensure the Low Conscientiousness story validated as neutral on Extraversion.

^5^All categories were significant with *p* < 0.001, with F_(4, 290)_ = 9.53 for Advice, F_(4, 290)_ = 39.57 for Celebration, F_(4, 290)_ = 11.45 for Empathy, F_(4, 290)_= 6.08 for Supported.

^6^For example, as shown in Table 3 for score 1, participants in total used 44 Advice statements for the Low Con case, resulting in an average per participant of 1.47 statements (i.e. 44/30).

^7^In Saccardi and Masthoff (2023), two algorithms were presented, one based on subsets and one on mediums, which differed slightly. We opted here to use the subset one, as it was simpler (making fewer adaptations to ES level) and had more statistical validity.

^8^And several cases that were already covered under the previous point.

^9^In fact, looking at the details in Saccardi and Masthoff (2023), C2 was used as often as C6, and only included in their algorithm to make it similar to the score 5 case.

^10^It is even valuable when the personality of the rater is not known, as the medium cases in the algorithm can be used. This has already been done and the adapted survey is currently being used in a course.

^11^The medium cases of the algorithm were used for this, given the lack of personality data.

^12^Or what we expect to be the best support, in case we decided to compare adaptive to non-adaptive support.
